# Postoperative Benefits of Soft Tissue Wrist Arthroscopy: Retro- and Prospective Analyses of Outcome Measures

**DOI:** 10.3390/jcm13082280

**Published:** 2024-04-15

**Authors:** Wolfram Demmer, Emanuel Meyer, Denis Ehrl, Elias Volkmer, Bernhard Lukas, Nina F. Knie, Riccardo E. Giunta, Nikolaus Wachtel

**Affiliations:** 1 Division of Hand, Plastic and Aesthetic Surgery, University Hospital LMU, 81377 Munich, Germany; 2 Clinic of Hand Surgery, Helios Klinikum München West, 81377 Munich, Germany; 3 Center for Hand Surgery, Microsurgery and Plastic Surgery, Schoen Clinic Munich Harlaching, 81547 Munich, Germany

**Keywords:** wrist arthroscopy, objective and patient-reported outcome measures, PROMs, handsurgery

## Abstract

**Background**: Elective soft-tissue-only wrist arthroscopy is a standard procedure in hand surgery for the diagnosis and treatment of wrist pain. A number of pathologies can be treated arthroscopically, with the aim of pain reduction and improved wrist mobility. A postinterventional evaluation of the treatment using objective and patient-reported outcome measures (PROMs) allows for an evidence-based statement about the benefits of soft-tissue-only wrist arthroscopy. **Methods**: A dual-center study combining retro- and prospective clinical analyses of patient outcomes after soft-tissue-only wrist arthroscopies was performed. The data were collected at two hospitals with departments specializing in hand surgery. The outcome was measured by assessing the range of motion of the wrist and its manual strength, as well as PROMs, including Disabilities of the Arm, Shoulder and Hand (DASH) scores. **Results**: A total of 154 soft interventions met the study criteria and could be included. Seven months after the elective soft-tissue-only wrist arthroscopies, mobility improved significantly for active extension and flexion, as well as the ulnar and radial abduction of the wrist. The grip strength also improved significantly, by an average of 6 kg, during this period. The DASH score improved significantly, from 35 points to 14 points. Additionally, wrist pain at rest was reduced significantly. **Conclusions**: After elective soft-tissue-only wrist arthroscopy, patients showed an overall functional improvement in their wrist, with a significant reduction in pain and improvement of mobility and grip strength. This study emphasizes the importance of wrist arthroscopy as a successful treatment option for soft tissue pathologies of the wrist.

## 1. Introduction

Due to its minimally invasive nature and low complication rates, arthroscopy of the wrist has become the gold standard for the diagnosis as well as treatment of a large number of pathologies of the wrist [1,2]. These include the staging of injuries of the carpal cartilage or the triangular fibrocartilage complex (TFCC), as well as the stability of the carpal ligaments [2,3,4,5,6,7]. Moreover, wrist arthroscopy is routinely used for the treatment of soft tissue pathologies of the wrist, such as the excision of ganglion cysts, synovectomy, the debridement of degenerative cartilage and TFCC tears or the capsular refixation of traumatic TFCC tears [1,2,5,6].

Interestingly, the data on outcome measurements after wrist arthroscopies are still limited, despite their widespread use [8]. Moreover, the success of these procedures is significantly influenced by postoperative patient satisfaction. A postoperative assessment should therefore aim to not only evaluate objective criteria but also obtain a comprehensive view of the disability from the patient’s perspective [9,10]. Indeed, patient-reported outcome measures (PROMs) are of growing importance in decision-making in healthcare policies [11]. In contrast to objective outcome measures, PROMs focus on the patient’s functional abilities, capacity to resume normal daily activities and ability to return to work. The most commonly used tool to assess PROMs in wrist pathologies is the Disabilities of the Arm, Shoulder and Hand (DASH) score, which characterizes the overall functionality of the upper extremity and shows good reliability, validity and responsiveness in psychometric studies [12,13]. Other well-established PROMs include the Numerical Rating Scale (NRS) for pain assessment and overall patient satisfaction [14,15,16,17].

Considering the growing wide-spread use of wrist arthroscopy and the still-limited data on objective as well as subjective (i.e., PROMs) outcome measurements, this study aims to assess the postoperative benefits of wrist arthroscopy in a dual-center study. Only soft-tissue-only wrist arthroscopies were included in the assessment to allow for a better comparison of the postinterventional outcomes.

## 2. Materials and Methods

### 2.1. Study Design

The study was designed as a dual-center study combining retro- and prospective clinical analyses of patients before and after soft-tissue-only wrist arthroscopies were performed to assess the postinterventional outcomes of the surgical procedure. The study was approved by the ethics committee of the Medical Faculty of LMU Munich, Germany (approval number: 19-530). The centers enrolled in the study were the LMU Hospital and Schoen Clinic, both located in Munich, Germany. Arthroscopies were conducted by 7 and 12 different surgeons, respectively.

Retrospective cases of patients who had received an arthroscopy of the wrist within the period of March 2019 to February 2020 were included. The code for wrist arthroscopy in the German Operation and Procedure Classification System (OPS) was used to identify appropriate cases.

This study’s prospective phase took place between February and September of 2020. The inclusion criteria were elective arthroscopies focusing on soft-tissue-only disorders without the involvement or treatment of bony structures and without complex side procedures. Patients with preoperative infection, preoperative antibiotic treatments, re-operation during the study period, pregnancy, or an insurmountable language barrier were excluded from the study.

The prospective cohort was examined both before arthroscopy and during a seven-month follow-up; the retrospective cohort was only assessed at their last designated follow-up, 7 months postoperatively. Objective parameters as well as subjective PROMs were analyzed. In addition, retrospectively recruited patients were asked to provide certain PROMs retrospectively for their preoperative time period (recall survey).

### 2.2. Data Collection/Recall Survey and Validation

The prospective patient cohort was assessed preoperatively, and follow-up assessments were conducted at 2 weeks, 8 weeks and 7 months after the intervention (endpoint). Objective and subjective (PROMs) outcome measurements were evaluated at those times.

The patients that were investigated retrospectively were assessed 7 months after their wrist arthroscopy in a validated recall survey. Previous studies have demonstrated the significance of recall DASH scores for hand surgery patients [18,19]. The patients were asked to describe the resting and maximum pain that they experienced before arthroscopy. Likewise, they were asked to complete the DASH questionnaire regarding the period immediately before arthroscopy.

Thus, for all patients, objective outcome measurements and PROMs were examined preoperatively (including recall surveys for the retrospective cohort) and 7 months postoperatively. Additionally, (for the prospective cohort) grip strength and ROM were assessed 8 weeks postoperatively and the DASH score, the NRS, and patient satisfaction were assessed 14 days and/or 8 weeks postoperatively.

### 2.3. Methods of Measuring Objective Outcomes

The active ROM of the wrist was measured using a full-circle goniometer and standardized positioning of the arm [20,21,22,23]. The result of each movement was documented in degrees of deviation from the neutral position of the wrist. Additionally, the passive ROM was assessed for extension and flexion in the same manner.

The grip strength was measured using a hydraulic hand dynamometer (Saehan corp., Changwon-si, Republic of Korea) [24]. After a proper adjustment of the grip seize to the patients’ hand, they were asked to grip the measuring device as firmly as possible. The examination was carried out in a standardized fashion and with a 90° flexion of the elbow. The measurements were taken, in triplicate, in kg.

Sensitivity was assessed using the static two-point discrimination test (s2PD) as described previously [25,26,27]. Sensitivity was measured at the area of the arthroscopy portals.

### 2.4. Methods of Measuring Patient-Reported Outcomes (PROMs)

The DASH score is a region-specific PROM that assesses the symptoms and functional status of the shoulder, arm and hand [28]. The questionnaire consists of 30 questions, generating a score ranging from 0 (no disability) to 100 (most severe disability). Additionally, two optional modules were answered by the patients. These specifically assess disabilities in terms of sport/music and work assessment [29,30]. In this study, the German version of the questionnaire was used [30].

Moreover, patients were asked to rate the pain in the affected hand at (1) absolute rest (minimal resting pain) and at (2) maximum exertion using a numerical rating scale (NRS) ranging from a minimum of 0 to a maximum of 10 (maximum pain).

To evaluate postoperative patient satisfaction a 5-point Likert scale was used. Patients were asked to rate their perceived overall function of their operated wrist after arthroscopy (Table 1). At 8 weeks and 7 months postoperatively, patients were asked to rate the overall change in the condition of their hand/wrist due to the procedure.

### 2.5. Statistical Analysis

For statistical evaluation, the GraphPad Prism (Version 8.0.2.; GraphPad Software, Inc., San Diego, CA, USA) was used. Groups that were matched were checked for normal distribution by *D’Agostino and Pearson-tests*. To compare the different groups *Student’s t-test* was used for normally distributed samples and the *Wilcoxon signed rank test* was used for non-parametric samples. Nominal or ordinal scaled values were checked for significant differences by *Fisher’s exact test*. For more than two variables, the *Chi-square test* was used. A *p*-value of <0.05 was considered statistically significant.

## 3. Results

### 3.1. Demographic Data

**Figure 1:**
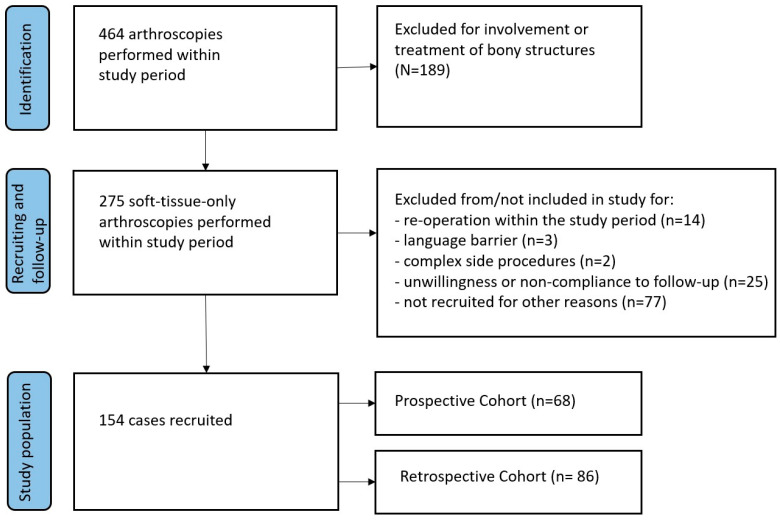
Study flow chart.

In the designated study period, a combined total of 464 arthroscopies were carried out in both hospitals, with 275 of them being soft-tissue-only arthroscopies. Among these, 154 patients met the inclusion criteria, which included a willingness to participate in the study, being older than 18, soft-tissue arthroscopy only, and no other exclusion criteria such as re-operation within the study period, a language barrier, complex side procedures, unwillingness, or non-compliance with follow-up. In total, 68 cases (44.1%) were recruited prospectively, and 86 cases (55.8%) were recruited retrospectively (Figure 1).

**Figure 2:**
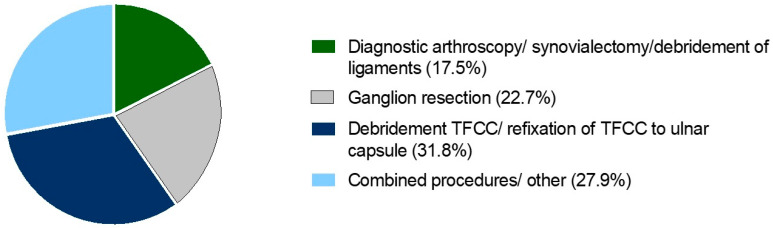
Distribution of arthroscopic procedures included in the study (rounded up to the first decimal place). *n* = 154.

Of the 154 patients included in the study, 89 were female (57.8%) and 65 were male (42.2%). On average, the patients were 37.1 (±15.8) years old. The majority of arthroscopies focused on pathologies of the TFCC (31.8%) (Figure 2).

The dominant hand was involved in 54.5% of wrist arthroscopies. In 49.9% of cases the wrist disorders had occurred post-traumatically, and in 16.2% of cases the operated hand had already undergone previous surgery. The mean immobilization period after the operation was 11.1 days (±8.6). Postoperative physiotherapy and occupational therapy were conducted for 74.0% of the patients.

For a total of 150 patients, objective outcome measurements and PROMs were examined preoperatively (including recall surveys for the retrospective cohort) and 7 months postoperatively. Additionally (for the prospective cohort), grip strength and ROM, as well as DASH score, the NRS and patient satisfaction with the procedure, were assessed at 14 days and/or 8 weeks postoperatively (*n* = 64 and *n* = 68) (Table 2).

The preoperative data obtained through the recall survey of the retrospective cohort were internally validated using the prospective group of the study, as described previously [18,19]. No significant difference could be observed between the preoperatively recorded DASH scores and the retrospectively collected data in the recall survey after 7 months (0.07 points; *p* = 0.956). The mean recall error was 7.02 points, which is less than the Minimal Clinically Important Difference (MCID) of 10.83 to 15 points, as reported in the literature [31,32]. For maximum wrist pain on exertion, the 7-month recall assessment largely corresponded to the preoperatively determined values (as opposed to minimal resting pain, where there was a recall bias). The mean recall error was 1.08 points, which is less than the MCID of 1.6 to 1.9 points observed in hand surgery patients [33], thus proving the sufficient validity of their respective parameters. These findings were therefore incorporated into the evaluation on an equal basis with the data collected prospectively (Table 2).

### 3.2. Results of Objective Outcome Measurements

**Figure 3:**
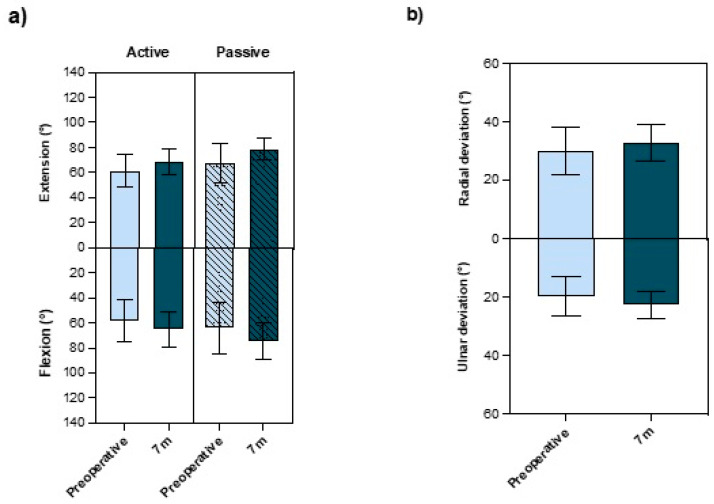
(**a**) Measurements of active and passive extension and flexion, as well as (**b**) active ulnar and radial deviation, pre- and 7-month postoperatively (prospective cohort). *n* = 47, bars represent means ± SD, individual values are presented as dots. Statistical differences were calculated using Student’s *t*-test (paired).

During the postoperative monitoring at the 8-week follow-up, we observed no significant changes in wrist ROM when compared to preoperative ROMs. Seven months after surgery, however, a significant improvement was observed in both extension and flexion, as well as in ulnar and radial deviation (Figure 3). Active extension and flexion increased by an average of about 7°. The passive ranges of motion showed an average improvement of 10° each. Ulnar and radial deviations increased by about 4 and 2°, respectively. Pronation and Supination showed no deficit before and no change after the operation (both 89°).

**Figure 4:**
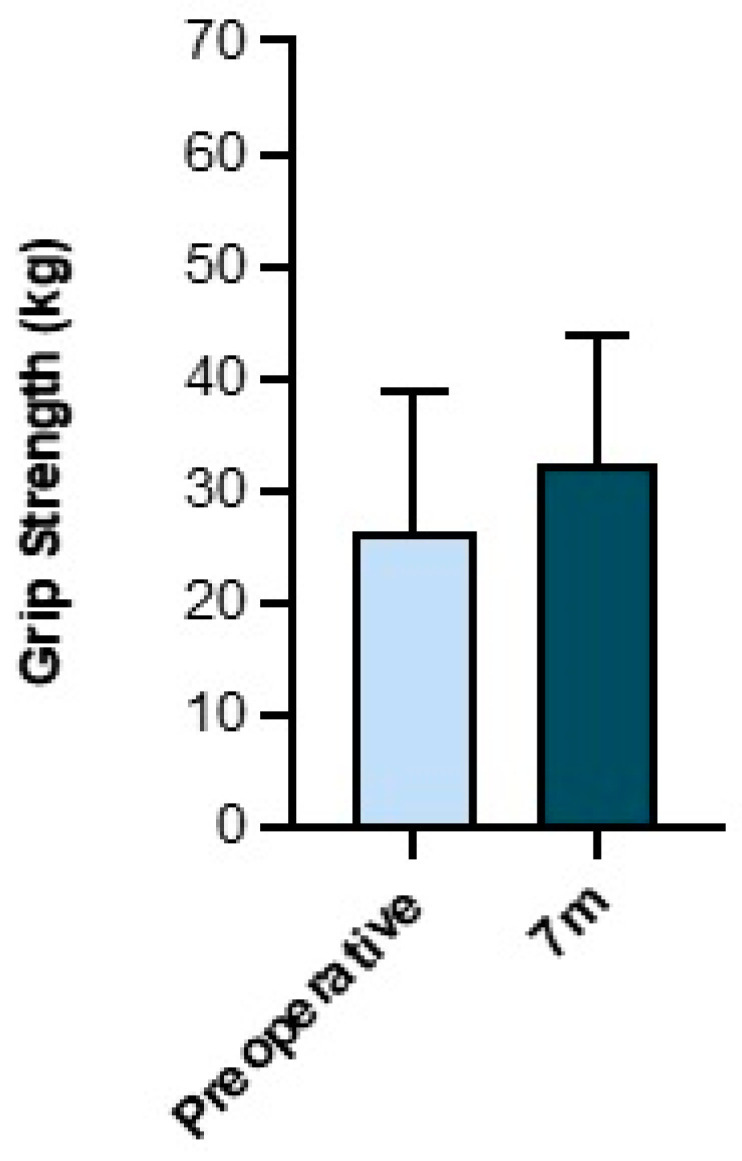
Grip strength in comparison pre- and 7-month postoperatively, in kg (prospective cohort). *n* = 47, bars represent means ± SD, individual values are presented as dots. Statistical differences were calculated using Student’s *t*-test (paired).

At 8 weeks after their intervention, patients showed no significant difference in grip strength. However, seven months after surgery, a significant improvement could be demonstrated when compared to the preoperative data. On average, the increase in strength amounted to approximately 6 kg (*n* = 47) (Figure 4).

Patients exhibited a two-point discrimination of 2.1 (±0.7) cm in the dorsum of the hand and wrist, the intended location for the surgery, prior to the operation (*n* = 28). There was no significant difference postoperatively (2.0 ± 0.5 cm), when compared to their preoperative status, indicating an unchanged static discrimination ability in that area (*n* = 28). These results were comparable with the 7-month data of the retrospective group (1.9 ± 0.5 cm, *n* = 46).

### 3.3. Results of Patient-Reported Outcome Measures (PROMs)

**Figure 5:**
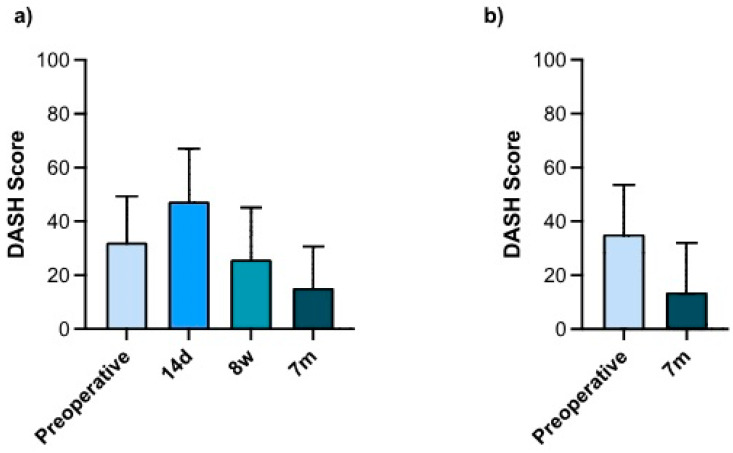
(**a**) DASH scores during the study period (prospective cohort). *n* = 51. (**b**) Preoperative and 7-month postoperative DASH scores (prospective and retrospective cohort). *n* = 148. Bars represent means ± SD; individual values are presented as dots. Statistics: (**a**) Friedmann Test + Dunn’s multiple comparisons post hoc test; (**b**) Wilcoxon signed rank test (paired).

The DASH score was monitored closely during the postoperative course of the prospective cohort. In the first postoperative control, 14 days after the intervention, the DASH score showed a worsening from 32 (±17) points preoperatively to 47 (±20) points postoperatively. Subsequently, the trend was reversed, and we observed a significant improvement of the DASH score seven months after surgery, with a decrease of over 50% to 15 (±15) points (*n* = 51). The same trend was observed when including the retrospective recall data. Here, the score improved from 35 (±18) to 14 (±16) points (*n* = 148) (Figure 5).

The postoperative course of the two optional DASH modules for music/sport and work mirrored the trend of the overall DASH score. Seven months after surgery, the score for both modules was significantly reduced, from 68 and 49 preoperatively to 32 and 17 points for the music/sport (*n* = 33) and work (*n* = 136) modules, respectively. However, we observed a slower recovery of the scores of both modules compared to those of the overall DASH questionnaire, with higher scores at the visits 14 days and 8 weeks after surgery.

**Figure 6:**
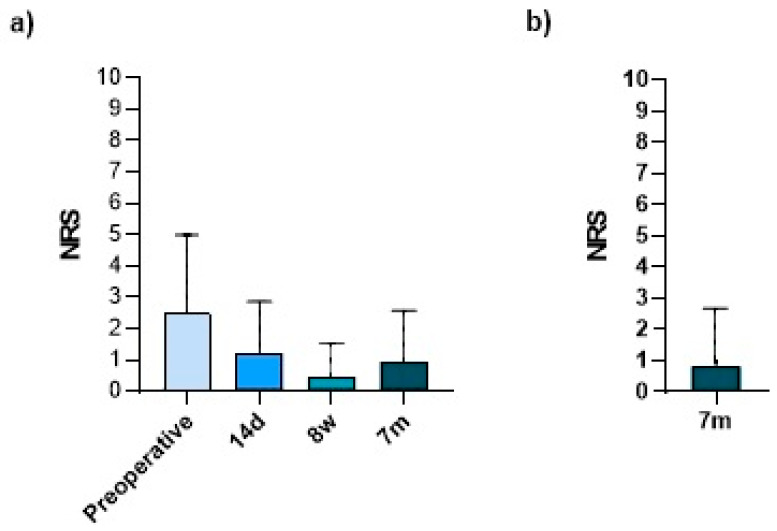
(**a**) Minimum resting pain on exertion in the postoperative course of the prospective cohort (*n* = 57) and (**b**) minimum resting pain in the retrospective cohort after 7 months (*n* = 86). Bars represent means ± SD; individual values are presented as dots. Statistics: (**a**) Friedmann Test + Dunn’s multiple comparisons post hoc test.

Before arthroscopy, patients reported an average minimal resting pain of 2.5 out of 10 (±2.5) for the affected hand. In their postoperative course, the intensity of their resting pain decreased by more than 50%, reaching 1.0 out of 10 points (±1.6) after 7 months (Figure 6a). In the visits 14 days and 8 weeks after surgery, scores of 1.2 (±1.6) and 0.48 (±1.0) were reported by patients in the prospective cohort (*n* = 57). Their results after 7 months were equal those of the retrospective cohort (0.8 ± 2.0; *n* = 86) (Figure 6b).

**Figure 7:**
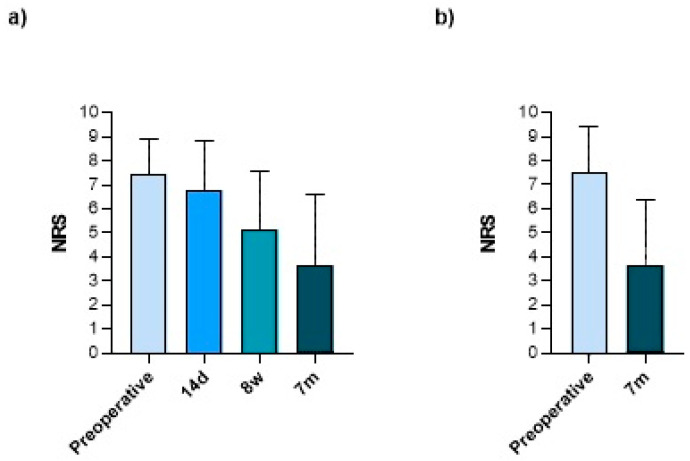
(**a**) Maximum pain on exertion in the postoperative course of the prospective cohort (*n* = 59) and (**b**) comparison of pre- and postoperative maximum pain on exertion of the prospective cohort and the retrospective cohort (*n* = 149; including recall surveys). Bars represent means ± SD; individual values are presented as dots. Statistics: (**a**) Friedmann Test + Dunn’s multiple comparisons post hoc test, (**b**) Wilcoxon signed rank test (paired).

A similar result was observed for the maximum pain on exertion. The patients reported a score of 7.5 (±2.0) preoperatively (*n* = 149), 6.8 (±2.1) after 14 days and a score of and 5.1 (±2.4) 8 weeks after surgery (both *n* = 59). The score reached, with 3.7 (±2.9), its lowest point seven months after surgery (*n* = 149) (Figure 7).

**Figure 8:**
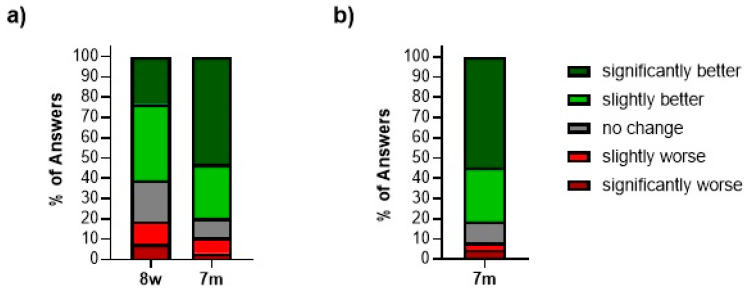
Subjective change after wrist arthroscopy (**a**) in the prospective cohort during their postoperative course (*n* = 64) and (**b**) in the retrospective cohort after 7 months (*n* = 87). Statistical differences were calculated using a Chi-square test.

Patients in the prospective cohort were asked to rate their perceived overall function of their operated wrist after arthroscopy (Table 1). The majority felt an improvement after the procedure. At eight weeks, 61% reported that the overall condition of their hand was “slightly” or “significantly better”. Seven months after surgery, 53% reported a “significantly” and 27% a “slightly better” function of the wrist (*n* = 64). These findings were supported by the evaluations of the retrospective cohort at their 7-month follow-up, which showed almost identical results (*n* = 87) (Figure 8).

## 4. Discussion

For variety of soft tissue wrist disorders, arthroscopic procedures have become the gold standard for therapy [1,2,5,6]. However, the literature on the postoperative outcomes of these procedures is surprisingly scarce or predominantly focuses on specific pathologies only [8]. This study was therefore conducted to allow for a valid outcome measurement (objective results as well as PROMs) of soft-tissue-only arthroscopies of the wrist. Our prospective cohort reflects the development of the parameters investigated over a period of 7 months. Their retrospective data, together with data from the recall survey, underline the implications of our findings.

We observed a significant improvement in the objective outcome measurements gathered in this study. Following arthroscopy, the ROM of the wrist was significantly improved (Figure 3). Also, the grip strength of the operated hand significantly improved by approximately 6 kg at the 7-month follow-up evaluation (Figure 4). These findings are comparable with the results of other studies [34,35,36,37,38]. Moreover, we observed no change in two-point discrimination at the surgical site on the dorsum of the wrist, confirming the low risk of nerve damage associated with wrist arthroscopy [39,40].

The DASH score, as a general indicator of upper extremity function, is one of the most commonly used and well-established PROMs used to assess the status of the wrist and upper extremity as perceived by the patient [12,13]. In this study, a significant reduction of this score was observed when comparing preoperative and postoperative values (Figure 5). Interestingly, we observed an initial deterioration of the score at the 14-day examination. This is most likely due to surgical trauma and immobilization due to the procedure. Over the remaining course of the study and at the final examination at 7 months post operation, a consecutive 50% decrease in the score was observed, indicating a significant improvement in wrist function. No clear benchmarks for the interpretation of DASH scores have been established. However, previous studies consider a score of 0 to 10 a “very good” outcome; with a score of 0 to 29, most patients no longer “consider their upper limb disorder a problem” [29,41]. In this study, we observed an average postoperative score of 15 points at 7 months after surgery in the prospective cohort. Patients initially scored an average of 32 points or higher (when including the recall survey of the retrospective cohort). The arthroscopic procedures in this study therefore resulted in the good to very good function of the upper extremity (Figure 5).

Only a relatively small number of patients completed the music/sport module. Nevertheless, the slower recovery of these scores compared to those of the overall DASH questionnaire is consistent with the descriptions in the literature [42]. This can be seen as an indication of the high demands placed on the wrist during these activities. Equally, the slower recovery of the work module compared to the overall DASH correlates with the postoperatively delayed increase in hand strength. The reason may be linked to the patient’s gradual involvement in manual tasks. At the end of the follow-up period, the scores of both optional DASH modules showed about the same relative score decrease as the overall DASH (Figure 5).

For the prospective cohort, a significant reduction in resting pain was already observed at the 14-day follow-up, reaching its lowest point 8 weeks postoperatively. A slight increase, albeit not significant, in persistent resting pain was observed after 7 months. Most likely, this can be attributed to postoperative immobilization as well as analgetic medication. Our findings for pain on exertion showed an average reduction of over 50% in the final follow-up examination compared to preoperative conditions. However, residual pain on exertion remained at 3.7 out of 10 on the NRS. These findings are validated by other studies that showed a significant reduction in pain after a soft-tissue arthroscopy of the wrist [43,44,45].

At the final examination 7 months after arthroscopy, 80% of all patients expressed an improvement when questioned about the status change of their affected wrist (Figure 8). Taken alone, this subjective statement of patient satisfaction has little significance in determining the success of a surgical procedure. However, when combined with the measurable and significant improvements shown in this study, it underlines the benefit of soft-tissue-only arthroscopy across the study collective and is comparable to other studies that report a patient satisfaction of up to 86% after similar procedures [35,43].

This study demonstrates that elective wrist arthroscopy successfully treats soft tissue pathologies of the wrist with significant measurable improvements in wrist mobility and grip strength, as well as a significantly increased overall function of the wrist and reduction in the pain perceived by the patient. Thus, approximately 80% of the patients who underwent wrist arthroscopy achieved a “good” to “very good” outcome, according to previous studies [29,41]. However, in particular, the objective outcome parameters that were examined in this study changed significantly between the eight-week and seven-month visits (Figure 3 and Figure 4). Patients should therefore be prepared before surgery to expect an improvement of their symptoms primarily between 8 weeks and 7 months after the procedure.

Moreover, while the DASH questionnaire demonstrates good reliability, validity and responsiveness, the test has its limitations, as it does not differentiate between the individual potential functional disorders of the joints themselves, but only assesses disorders of the upper extremities as a whole [13]. Other limitations of this study arise primarily from the chosen cohort under study. Thus, recall bias (for the retrospective cohort), as well as facility-dependent effects, cannot be completely ruled out. Future studies should therefore validate our results and the findings of others in large-scale prospective studies with a multi-center approach.

## 5. Conclusions

Despite its relatively small sample size, this study clearly demonstrates the benefits of soft-tissue-only arthroscopy for patients. Postoperatively, improvements were objectively observed in terms of enhanced wrist mobility and grip strength, as well as in patient-reported outcome measures (PROMs), such as the DASH score, indicating subjective enhancements in wrist function and reductions in pain. In the seven-month follow-up, 80% of the patients reported an improvement in their wrist condition.

## Figures and Tables

**Table 1:** 5-point Likert scale.

**Subjective Change in the Wrist Due to the Surgery**	**Significantly Worse** **□**	**Slightly Worse** **□**	**No Change** **□**	**Slightly Better** **□**	**Significantly Better** **□**

**Table 2:** Time frame and follow-up rate of examinations for prospective and retrospective cohorts.

	Pre-OP	14 Days	8 Weeks	7 Months
Objective Outcome measures				
Range of Motion	Prospective (*n* = 58)	-	Prospective (*n* = 27)	Prospective (*n* = 52) Retrospective (*n* = 73)
Grip Strength	Prospective (*n* = 59)	-	Prospective (*n* = 25)	Prospective (*n* = 54) Retrospective (*n* = 73)
Sensitivity Testing	Prospective (*n* = 36)	-	-	Prospective (*n* = 49) Retrospective (*n* = 46)
Patient-reported outcome measures (PROMs)				
DASH Score	Prospective (*n* = 63) * Retrospective (*n* = 86)	Prospective (*n* = 59) *	Prospective (*n* = 56) *	Prospective (*n* = 63) * Retrospective (*n* = 86)
Pain Assessment (NRS)	Prospective (*n* = 66) Retrospective (*n* = 86) **	Prospective (*n* = 64)	Prospective (*n* = 68)	Prospective (*n* = 64) Retrospective (*n* = 86)
Patient Satisfaction	-	-	Prospective (*n* = 68)	Prospective (*n* = 64) Retrospective (*n* = 86)

*, partly including modules regarding sport/music and work; **, only wrist pain on exertion.

## Data Availability

The authors confirm that the data supporting the findings of this study are available within the article.
